# A Memorial of Certain Researches on the Perspiratory Vessels of the Skin

**Published:** 1831-07

**Authors:** 


### THE SKIN.

### *A Memorial of certain Researches on the Perspiratory Vessels of the Skin.* By Dr. Hake.

There are men who study anatomy as a means of directing their movements in the treatment of disease; and there are philosophers who do so with an additional motive, the love of all related to so fine and curious a science. Since it alike concerns the physiologist and physician, the structure of the skin has occupied much attention; to its anatomical history many great names are affixed. While the skin, as a system, ranks not lowly in the degrees of vital importance, it is by far the most beautiful of all the living textures, not only as to configuration, it being the final stroke to human symmetry, but from its fineness and softness of texture, and purity of colour.

It is very well understood that the exterior surface of the skin is perpetually moistened by the transpiration of a saline fluid: during the spring of 1830, I first observed the perspiration on the points of the fingers, to follow a determinate arrangement, of which the investigation led to the conclusions which follow: at some of them, however, the French anatomist, Beclard, had previously arrived. "The surface of the skin," says Beclard, "presents small wrinkles peculiar to the epidermis, in the palms and soles: these are prominent lines, separated by other depressed lines, running in various and winding directions, and which are formed by rows of papillæ." In my notes I have preserved an account which to the above is dissimilar but in language, the facts being the same; but there are some minute points besides, which Beclard probably saw, without copying down: they are as follows.

1. On submitting the base of the ridge, or prominent line, to the micrometer, its width was found to occupy one fiftieth of an inch of space; whilst that of the groove, or depressed line, occupied only one three-hundredth part of an inch.

2. The furrows are concave from side to side, and from their edges, which are distinct, arise the sides of the ridges which meet above at an angle, and thus, with their attached base, produce the form of a prism. The width of either side is about one ninety-sixth part of an inch, but the sides themselves are differently occupied: the one being covered by papillæ, which are one fiftieth of an inch remote from each other, and arranged in a regular series; the other side being occupied by superficial grooves, each of which passes from the porous aperture of a papilla, and descends into the groove: by the latter arrangement, the globule of perspiration, which arises through the pore of the papilla, passes also into the groove, and thus is equally diffused over the surface.

3. By dissecting away the ridge, layer by layer, to the level of the furrow, each new surface presents an opening, which is perpendicular to the spot formerly occupied by the mouth above.

On first observing these phenomena, there are three circumstances apt to cause deception: 1. Owing to the inclination of the sides of the ridges, the papillæ may appear to be in the furrow. 2. The papillæ, if viewed from the sides which they occupy, seem to be on the summits of, or rather to constitute, the ridges. 3. If the ridges be viewed from the side on which the grooves are, the whole cuticular surface, owing to the meeting of grooves and furrows, looks reticulated.—July 1830.

On similar matters, other notes have collected between July and the following February: they relate to the skin which covers the dorsum of the finger, the structure of which is far more difficult to distinguish, intense lights and exquisite lenses having failed to develope its true order, during many severe investigations. The first memorandum is to the following effect: "The cutaneous surface is, on the back of the fingers, traversed by grooves of many sizes, which frequently, and in all directions, cross each other, so as ultimately to leave only so many spots of uninterrupted surface. When, in order to find out the ultimate design of all this, a powerful lens is preferred, it is soon discovered that the former structure is so deformed as to yield no information: in fact, it will not bear the action of a high magnifier; but a moderate lens will, without any distortion, sufficiently enlarge the field of nature. If a mild perspiration be present, glistening particles of fluid are here and there observed, generally at equal distances from each other; and thus they follow the courses of the grooves, not occupying their cavities, but the summits of their sides: this is the more remarkable on the dorsum of the second phalanx of the fingers, but consequent on the frequency of the decussation of grooves, even there is often confused. A superficial furrow may be occasionally traced, extending from a pore to a groove, but such is only to be regularly seen on the palms. The pores vary in size; those of the palmar surfaces being larger than on the backs of the fingers, and the latter than elewhere: were I asked the reason of this variation, my experience would lead me to say, that it was instituted to give passage to a fluid more or less impregnated with saline matter."

A memorandum succeeds, which throws some light on the structure of the pores. "On the back of the first phalanx, the texture of the cuticle is fine and translucid: observing which, I deemed it probable that a microscopic examination might be attended with a useful result: by dint of care and labour, I was delighted with the sight of a few small and exquisitely attenuated vessels, of a red colour; they were directly under the cuticle, to all appearance, and, having emerged, they seemed to creep a little way, then open with red mouths, and become continuous with the epidermis. As far as could be distinguished, each vessel became first visible at a little distance from one pore, and, after a short course, opened to form the next." It may here be mentioned, that I have witnessed these phenomena several times since the above account was written, and that at this moment I have again confirmed the statement.

Let us now pass on to some more important observations, bearing the date of April 1831.

After having soaked the hand in very hot water, and again dried it, I examined the dorsal surface with the weakest power of the simple microscope, and found it regularly studded with aqueous globules, which, while the parts remained swoln, were no sooner removed than replaced. By a sudden and undesigned movement of the lens, a reticular work became visible, which seemed to be of vessels: although this appearance was as evident as if of far greater size, I doubted its reality, lest it should consist of so many grooves, which, in a peculiar light, might have the aspect of elevation; but, on a comparison with such as I knew to be only grooves, the distinction was marked. I have mentioned that there are left between the grooves only so many spots of uninterrupted surface: on these it is that the network above mentioned exists: it is so distinct on the joints as to become visible, by practice, to the naked eye. On these perspiratory vessels I observed numerous projecting points, resembling truncated ramuscules of the prime branch. Under the light requisite to produce these phenomena to vision, the network has a beautiful blue colour. At the summits of the sides of grooves (which on some joints subdivide in the form of vessels,) it is that the vascular elevation is most conspicuous: one vessel I saw running down the side of a groove. The anastomoses of these vessels seem very frequent, and at different angles, especially at ninety, sixty, and one hundred and twenty degrees. At the apex of each papilla which is given off, the pore was visible, if not occupied by sweat, and not of a red colour, like that discovered near the roots of the nails."

Every one knows that the epidermis at the sides of the nails is much thicker than elsewhere. It was with a view to ascertain whether, and how, the pores are continued downwards, that I removed, with scissars, many layers of cuticle from those parts, and was astonished to see how well, not only the pores, but the ridges and grooves, maintained their characteristics: however, when the epidermis, instead of being cut, is peeled off of the palms, the layer which becomes exposed is precisely similar to that which is removed. But a fact, which I at that time observed, has since often attracted my attention, for I conceive it to be important, although simple: however dry the skin may be externally, as soon as a layer is removed, the pores of the new surface are occupied by sweat. To account for the occasional aridity of the skin, hypothesis has almost, of necessity, urged a spasm of the pores, and sudorifics have been said to have the property of relaxing the contraction. The above experiment demonstrates the point in which the spasm resides.

Much has been said concerning insensible perspiration. It is true that if the hand be presented to a mirror, its polished surface becomes tarnished; but this is owing to spontaneous evaporation from the excreted fluid. I have watched the progress of perspiration often, and always seen it to arise in a liquid state: nor is there sufficient reason to suppose an additional set of pores for an insensible exhalation.

The appearance which I have described as resembling a network of vessels, I believe to be an elevation of cuticle corresponding to the shape of the vessels beneath, which open at its surface. The thickness of the epidermis on the palms and soles precludes the possibility of discovering the arrangement of the vessels which lead to that membrane, but we may infer a regular and fixed distribution, from the harmonious order of the pores themselves.

Whether or not the perspiratory vessels were seen by KAAU and by Dr. HUNTER, it must be certain that they proceed from the dermis to the epidermis: from the researches of MALPHIGI and his successors, it can be no less certain that there are dermoid vessels which open to supply the fluid of, if not to form, the rete mucosum. These facts, had they been considered, might have served in explanation of the question, why the fluid effused beneath the cuticle from blistering does not escape through the pores, although BICHAT, as well as MECKEL and others, have used hypothesis for its solution. The cantharides, when applied to the skin, acts on the secreting vessels of the dermis, which pour out, in reply to their proper stimulus, a greater quantity of fluid on the cellular tissue which invests its exterior surface, under the denomination of rete-mucosum. How, then, can this fluid enter the porous vessels which are continued from the dermis, and open only on the surface of the cuticle?

Very accurate engravings might be made of the cutaneous pores; but it may be mentioned that, by substituting the palm of the hand for a plate, not an inelegant semblance may be struck off on paper. By impressing the joints of the fingers on clear glass, the sebaceous matter leaves an outline which becomes intelligible under a lens.

With regard to the mode of proceeding, it should be remarked that it is not through a strong, but rather through a weak light, that the objects on the skin are to be seen; that an egregious error is committed by those who use powerful magnifyers; and that those are not less mistaken who have searched for pores on the detached portions of skin, instead of examining living parts, during the performance of their functions.

49, *Grosvenor place; May* 16*th*, 1831.

---

